# Synergistic cognitive enhancement of repetitive transcranial magnetic stimulation with cognitive training in mild Alzheimer’s disease: preliminary results

**DOI:** 10.1002/alz.086785

**Published:** 2025-01-09

**Authors:** Juyoun Lee, Kyung Won Park, Kun‐Woo Park, Ae Young Lee

**Affiliations:** ^1^ Chungnam National University Hospital, Daejeon Korea, Republic of (South); ^2^ Dong‐A University College of Medicine, Busan Korea, Republic of (South); ^3^ Korea University, School of Medicine, Anam Hospital, Seoul Korea, Republic of (South)

## Abstract

**Background:**

Repetitive transcranial magnetic stimulation (rTMS) is a non‐invasive emerging tool to modulate brain activities and functional connectivity in various neuropsychiatric disorders. rTMS combined with cognitive training (rTMS‐COG) has been showing cognitive enhancing effects compared to those of placebo in mild Alzheimer’s disease (AD) in some previous studies. However, there is not much research to conclude how much each rTMS or COG contributes to therapeutic cognitive effects. This study aims to investigate the cognitive enhancing effect of rTMS‐COG in patients with mild AD.

**Method:**

This study is a prospective, randomized, double‐blind, placebo‐controlled multicenter study. All recruited patients with the mild stage of AD had amyloid deposition in positron emission tomography. Participants fulfilled the inclusion criteria, including a 21∼26 score on the Mini‐Mental Status Examination (MMSE) and the Clinical Dementia Rating (CDR) scale 1, or the Global Deterioration Scale 3. The treatment group (T) had 30 sessions of rTMS‐COG for 6 weeks. The active control group (S) had digital cognitive training without rTMS for 6 weeks. High frequency (10Hz) rTMS was applied on six cortical areas (bilateral dorsolateral prefrontal cortex, bilateral posterior somatosensory cortex, Broca’s area, and Wernicke’s area). The primary outcome measure of this study was the differences in the ADAS‐cog score changes between groups, and its difference of 24 weeks of treatment from the baseline.

**Result:**

Data from 20 patients (T:S 10:10) were used in the preliminary analysis. The mean values for the participants were 71.7 years of age, 10.5 years of education, and 20.2 ADAS‐cog score. There was no significant difference between the two groups' baseline epidemiological or cognitive characteristics. At 24 weeks, the ADAS‐cog score improved by 1.3 points from the baseline in T and worsened by 1.3 points in S, with a difference of 2.6 points between the two groups. The treatment group showed significantly better cognitive function compared to that of the active control group in the ADAS‐cog scores after 24 weeks from baseline.

**Conclusion:**

This preliminary outcome of the study suggests that rTMS‐COG treatment has significant positive cognitive therapeutic effects, and rTMS might take precedence over COG in terms of cognitive modulation in patients with mild AD.

